# A survey of canine tick-borne diseases in India

**DOI:** 10.1186/1756-3305-4-141

**Published:** 2011-07-19

**Authors:** Puteri Azaziah Megat Abd Rani, Peter J Irwin, Glen T Coleman, Mukulesh Gatne, Rebecca J Traub

**Affiliations:** 1 School of Veterinary Science, The University of Queensland, Gatton, Queensland, Australia; 2 School of Veterinary and Biomedical Sciences, Murdoch University, Western Australia; 3 Bombay Veterinary College, Parel, Mumbai, India; 4 Faculty of Veterinary Medicine, Universiti Putra Malaysia, Malaysia

## Abstract

**Background:**

There are few published reports on canine *Babesia*, *Ehrlichia*, *Anaplasma, Hepatozoon *and haemotropic *Mycoplasma *infections in India and most describe clinical disease in individual dogs, diagnosed by morphological observation of the microorganisms in stained blood smears. This study investigated the occurrence and distribution of canine tick-borne disease (TBD) pathogens using a combination of conventional and molecular diagnostic techniques in four cities in India.

**Results:**

On microscopy examination, only *Hepatozoon *gamonts were observed in twelve out of 525 (2.3%; 95% CI: 1.2, 4) blood smears. Using polymerase chain reaction (PCR), a total of 261 from 525 dogs (49.7%; 95% CI: 45.4, 54.1) in this study were infected with one or more canine tick-borne pathogen. *Hepatozoon canis *(30%; 95% CI: 26.0, 34.0) was the most common TBD pathogen found infecting dogs in India followed by *Ehrlichia canis *(20.6%; 95% CI: 17.2, 24.3), *Mycoplasma haemocanis *(12.2%; 95% CI: 9.5, 15.3), *Anaplasma platys *(6.5%; 95% CI: 4.5, 8.9)*, Babesia vogeli *(5.5%, 95% CI: 3.7, 7.8) and *Babesia gibsoni *(0.2%, 95% CI: 0.01, 1.06). Concurrent infection with more than one TBD pathogen occurred in 39% of cases. Potential tick vectors, *Rhipicephalus *(most commonly) and/or *Haemaphysalis *ticks were found on 278 (53%) of dogs examined.

**Conclusions:**

At least 6 species of canine tick-borne pathogens are present in India. *Hepatozoon canis *was the most common pathogen and ticks belonging to the genus *Rhipicephalus *were encountered most frequently. Polymerase chain reaction was more sensitive in detecting circulating pathogens compared with peripheral blood smear examination. As co-infections with canine TBD pathogens were common, Indian veterinary practitioners should be cognisant that the discovery of one such pathogen raises the potential for multiple infections which may warrant different clinical management strategies.

## Background

There is a relative paucity of studies into canine *Babesia*, *Ehrlichia*, *Anaplasma, Hepatozoon *and haemotropic *Mycoplasma *infections in India and most cases of canine tick-borne diseases (TBD) reported from the Indian subcontinent have been diagnosed by traditional methods using microscopic observation of microorganisms in stained blood smears [1]. This approach, based on morphological characteristics, does not permit reliable identification of the parasites. Serological approaches also have their limitations particularly as species-specific diagnosis is often required; both false positive [2,3] and false negative results [4] may confound interpretation. Since pathogenicity is known to vary significantly depending on the species of TBD pathogen [5], it is preferable to use molecular-based tools to investigate the clinical significance of canine TBD in India.

Babesiosis is an important disease of domestic and wild Canidae in Asia but the epidemiology of canine babesiosis in India is poorly understood. In a large study conducted in Chennai, *Babesia gibsoni *was reported with a prevalence of 0.1% [6] in client-owned dogs (n = 5,832) using bloods smear evaluation only. Other studies report 9% and 22% of dogs in Uttar Pradesh [7] and Assam [8], respectively, infected with *Babesia*, but the species of piroplasm infecting these dogs was not reported. The pathogenicity of *Babesia *is believed to vary in different regions of India and this is likely due to host factors and/or differences in the species present [1]. It is likely that both *Babesia vogeli *and *B. gibsoni *are co-endemic in India and the ticks *Rhipicephalus sanguineus *and *Haemaphysalis longicornis *are the putative vectors, respectively [5].

*Ehrlichia *is an alpha-proteobacterium belonging to the family Anaplasmataceae. Species that are able to produce infection in dogs are *Ehrlichia canis *(tropical canine pancytopenia), *Ehrlichia ewingii *(canine granulocytic ehrlichiosis) and *Ehrlichia chaffeensis *(human monocytic ehrlichiosis) [9,10]. The few studies investigating the prevalence of canine ehrlichiosis in India using conventional examination of stained blood smears have reported prevalences of 0.35% (n = 752) in Punjab [11], 18.9% in Nagpur (n = 238) [12] and 55% in stray dogs in Maharashtra [13]. One study utilizing an *E. canis*-specific nested PCR found 46/98 (46.9%) owned dogs in Chennai positive for *Ehrlichia *spp. compared to 19% by microscopy [14]. In this study however, amplicons were not sequenced to confirm the ehrlichial species and information about the clinical status of these dogs was also not reported.

Canine hepatozoonosis ranges from subclinical infections caused by *Hepatozoon canis *to severe, life-threatening disease caused by *Hepatozoon americanum *[15]. Transmission of *H. canis *to dogs occurs by ingestion of an infected tick, *R. sanguineus*, rather than tick bites [16]. Canine hepatozoonosis caused by *H. canis *has been reported most frequently as a subclinical infection in the north-west region of India, with a prevalence range of 3 to 9% in Punjab [17-19]. In other parts of the world, co-infection of *H. canis *with other infectious agents such as *Ehrlichia, Leishmania *and parvovirus is common [20-22].

Canine anaplasmosis is caused by intracellular rickettsial organisms of the genus *Anaplasma*. To date, two species have been identified as pathogenic in dogs; *Anaplasma platys *is the cause of canine infectious cyclic thrombocytopenia, and *Anaplasma phagocytophilum*, which parasitizes neutrophils, is zoonotic and causes granulocytic anaplasmosis in many countries in the northern hemisphere [22,23]. Single infections with *A. platys *are generally clinically unapparent but pathogenicity appears to be increased in co-infections [24].

Haemoplasmas are epierythrocytic parasites of mammals. Two species, *Mycoplasma haemocanis *and *Candidatus *Mycoplasma haematoparvum have been reported in dogs and the clinical effect of these microorganisms varies from asymptomatic infections to the induction of a severe haemolytic syndrome, especially in splenectomised or immunocompromised dogs [25,26]. Clinical disease caused by haemotropic *Mycoplasma *infections [27] and *A. platys *[28] have been reported in India but the prevalence and distribution of these pathogens remains largely unexplored.

This study was designed to investigate the occurrence and geographical distribution of canine TBD of veterinary and public health importance in India using a combination of conventional and molecular diagnostic techniques, and to examine associations between climatic and host-based risk factors and the presence of the various tick-borne diseases.

## Methods

### Animal data

**Figure 1:**
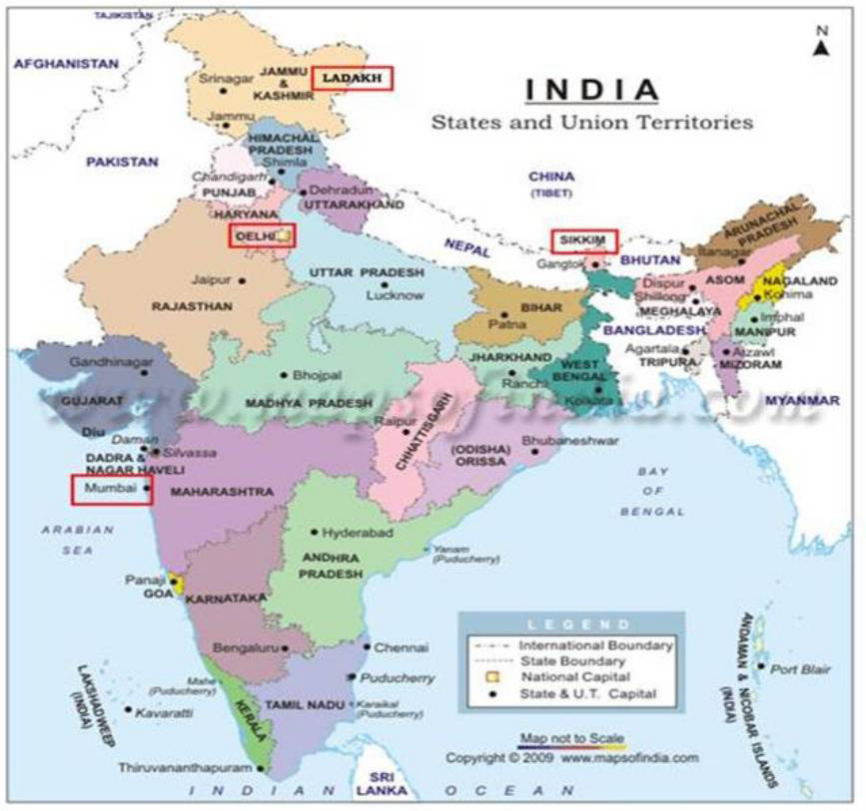
**Map of India**. Areas outlined in red rectangles indicate sampling locations.

Capillary and whole blood samples were collected from the cephalic and/or jugular veins of 525 dogs. Dogs were sampled at four sites, chosen to reflect the different climatic zones of India (Figure 1); Sikkim (subtropical highland) in northern West Bengal, Ladakh (montane region) in Jammu Kashmir, Delhi (monsoon-influenced humid subtropical region) and Mumbai (tropical region) [29], from June to September 2008. Sikkim and Ladakh are rural villages. To facilitate the fieldwork, collaborations were established with several locally-based partners; Vets Beyond Borders (VBB), Jeevaashram, Krishnaashram, Bombay Veterinary College (Mumbai) and In Defence of Animals India (IDAI). These organisations permitted us study access to stray and refuge dogs through their Animal Birth Control (ABC) and rabies vaccination programs, in which stray dogs are impounded, vaccinated, surgically neutered and released back to their original location. The refuge centres provide shelter, de-sexing and veterinary care where appropriate, for dogs that are either rescued from the streets or abandoned by their owners.

An estimate of each animal's age was made (based on dentition and body size) and classified as puppy (less than 6 months old), juvenile (between 6 months to 1 year old), adult (between 1 to 7 year old) and geriatric (more than 7 year old). Each animal's sex, body condition score and source (stray or refuge) [30] were noted, and the presence or absence of ticks was also recorded by searching the skin and hair coat of each dog for 1 minute. When present, a minimum of two ticks was collected and stored in 70% ethanol solution for later identification to genus level using Walker keys [31]. Body condition score (BCS) was determined using a 9-integer scale system [32]. All dogs were classified as apparently healthy or unwell based on their demeanour at the time of sampling, but a detailed clinical examination was not performed.

Blood smears were made from whole blood and buffy-coat preparations [33], air-dried and fixed in 100% ethanol and later stained with Giemsa for microscopic screening. Packed cell volume (PCV) was measured using a microhaematocrit centrifuge. Blood samples were also applied to Whatmans FTA cards^® ^for molecular-based screening. Microscope screening methodology and DNA extraction techniques have been described in detail previously [29].

### PCR assays and DNA sequencing

Nested PCR assays with primers targeting the partial region of the 18S rRNA gene were used for detection of canine piroplasm (*Babesia*, *Theileria*) species [34]. DNA amplification was performed under the conditions described by Jefferies and colleagues [34].

Touchdown PCR technique with ECA and HE3 primers [35] were used to amplify an approximate 400 bp fragment of 16S rRNA region of *E. canis *using conditions described by Gal and colleagues [36].

Forward primer, Platys and reverse primer EHR16SR [37] were used to amplify a partial region of the 16S rRNA gene of *A. platys *under the following conditions: 95°C for 5 min; 40 cycles of 94°C for 30 s, 55°C for 30 s, and 72°C for 90 s; then final extension at 72°C for 5 min.

For the detection of *Hepatozoon*, PCR was performed using primers HEP-F and HEP-R [38] under the following conditions: 95°C for 5 min; 40 cycles of 95°C for 30 s, 57°C for 30 s, and 72°C for 90 s; then final extension at 72°C for 5 min.

PCR detection of haemotropic *Mycoplasma *was performed using universal *Mycoplasma *spp. primers HBT-F and HBT-R [39] under the following conditions: 94°C for 5 min; 40 cycles of 95°C for 30 s, 60°C for 30 s, and 72°C for 90 s; then final extension at 72°C for 10 min.

The PCR products were run on a 2% agarose gel in 1 × TAE buffer at 100 V and visualised using GelDoc (Biorad). A randomly selected subset of products from 20% of the positive samples for each PCR protocol (Table 1) were purified using Qiagen spin columns (Qiagen) and sequenced using an ABI 3130xl Genetic Analyzer (Applied Biosystems) with Big Dye 3.0 chemistry. Sequences were edited and assembled using Finch TV (Geospiza Inc.) and compared to sequence data on GenBank using the Blast program (http://blast.ncbi.nlm.nih.gov/Blast.cgi) to confirm results.

**Table 1:** Primer sets for the PCR amplification and sequencing of canine TBD pathogens used in this study.

	Primers	Reference
***Babesia *species**	BTF1: 5'-GGC TCA TTA CAA CAG TTA TAG-3'	[34]
	BTR1: 5'-CCC AAA GAC TTT GAT TTC TCT C-3'	
	BTF2: 5'-CCG TGC TAA TTG TAG GGC TAA TAC-3'	
	BTR2: 5'-GGA CTA CGA CGG TAT CTG ATC G-3'	
***Ehrlichia canis***	ECA: 5'-AAC ACA TGC AAG TCG AAC GGA-3'	[36]
	HE3: 5'-TAT AGG TAC CGT CAT TAT CTT CCC TAT-3'	
***Hepatozoon species***	HEP-F: 5'-ATA CAT GAG CAA AAT CTC AAC-3'	[38]
	HEP-R: 5'-CTT ATT ATT CCA TGC TGC AG-3'	
***Anaplasma platys***	Platys: 5'-GAT TTT TGT CGT AGC TTG CTA TG-3'	[37]
	EHR16SR: 5'TAG CAC TCA TCG TTT ACA GC-3'	
***Mycoplasma *species**	HBT-F: 5'-ATA CGG CCC ATA TTC CTA CG-3'	[39]
	HBT-R: 5'-TGC TCC ACC ACT TGT TCA-3'	

### Statistical analysis

The prevalence and 95% binomial exact confidence intervals (CI) were calculated for the microscopy and PCR results for each TBD pathogen using Sourceforge.net^® ^(http://sampsize.sourceforge.net/iface/index.html). Association between canine TBD, host factors (age, gender, and source) and geographical location were evaluated using univariate analyses of odd ratios and their 95% confidence intervals using Chi-square test or Fisher's exact test for independence. Continuous data was analysed using one-way analysis of variance (ANOVA). Statistical significance was set at p ≤ 0.025. Multivariate logistic regression was used where data was substantial enough to quantify the association between the presence of vector-borne disease and host and climate variables after adjusting for other variables. Only variables significant at p ≤ 0.25 in the univariate analyses were considered eligible for inclusion in the multiple regression [40,41]. Backward elimination was used to determine which factors could be dropped from the multivariate model. The level of significance for a factor to remain in the final model was set at 5%. Statistical calculations were conducted using SPSS version 19.0 software (SPSS Inc., Chicago, IL, USA).

## Results

A total of 525 dogs, consisting of 42.1% intact females, 35.1% intact males, 12.3% neutered males and 10.5% neutered females were sampled; 77% were strays and 23% were shelter dogs. Upon visual inspection, 278 (53%) dogs were infested with ticks. The highest tick infestation was noted in Mumbai (prevalence 80%; n = 162) followed by Delhi, (prevalence 75.3%; n = 162); Sikkim (prevalence 17%; n = 101) and Ladakh (prevalence 11%; n = 100). A total of 832 ticks was collected and identified; the genus *Rhipicephalus *was found to be the most common dog tick present in this study followed by *Haemaphysalis*. The prevalence of tick genera by city are presented in Table 2.

**Table 2:** Prevalence of tick genera infecting dogs by sampling location

Tick genera	Mumbai (n = 417)	Delhi (n = 379)	Sikkim (n = 28)	Ladakh (n = 8)
***Rhipicephalus spp.***	100%	100%	44.4%	100%
***Haemaphysalis spp.***	0%	0%	55.6%	0%

Body condition scores (BCS) were recorded for 521 dogs. Scores were normally distributed; the mean ± SD BCS was 3.83 ± 1.12; the mode BCS was 3. Most dogs examined were adults (80%), followed by juveniles (9.2%), geriatrics (8.8%) and puppies (1.7%). All dogs were apparently healthy, except one that was found to be moribund due to a recent automobile accident. Packed cell volumes were reported for 441 dogs. The mean ± SD for PCV was 32.67% ± 8.9.

Blood smear examination of every sample was negative for *Babesia*, *Ehrlichia*, *Anaplasma *and haemotropic *Mycoplasma *spp., whereas *Hepatozoon *gamonts were observed in twelve (2.3%; 95% CI: 1.02, 3.58) samples. In contrast, using PCR, nearly half (261/525 or 49.7%; 95% CI: 45.4, 54.1) the dogs in this study were found to be infected with one or more canine TBD pathogens. All sequenced PCR amplicons were confirmed by comparison with published sequences on GenBank and matched with 99-100% homology. Of the 261 PCR positive dogs, 160 (61.3%; 95% CI: 55.1, 67.2) had single infections. Multiple infections with two or more canine TBD pathogens were found only in dogs from Delhi and Mumbai; 75 (28.7%; 95% CI: 23.3, 34.6) were co-infected with two, 22 (8.5%; 95% CI; 5.4, 12.5) with three and 4 (1.5%; 95% CI: 0.4, 3.9) with four species of canine TBD pathogens. Among 101 dogs that were positive for multiple infections, 50 (49.5%; 95% CI: 39.4, 59.6) were co-infected with *H. canis *and *E. canis*. The occurrence of canine TBD pathogens by molecular screening is summarised in Table 3 and Table 4.

**Table 3:** The prevalence (%) and 95% CI (lower, upper intervals) of canine tick-borne disease pathogens by city using molecular screening.

	Delhi (n = 162)	Mumbai (n = 162)	Sikkim (n = 101)	Ladakh (n = 100)
***Babesia vogeli***	8.6% (4.8, 14.1)	7.4% (3.9, 12.6)	2% (0.6, 8.4)	0% (0, 3.6)
***Babesia gibsoni***	0% (0, 2.3)	0% (0, 2.3)	1% (0.03, 5.4)	0% (0, 3.6)
***Hepatozoon canis***	38.3% (30.8, 46.2)	43.8% (36.1, 51.8)	0% (0, 3.6)	24% (16, 33.6)
***Ehrlichia canis***	39.5% (31.9, 47.5)	27.2% (20.5, 34.7)	0% (0, 3.6)	0% (0, 3.6)
***Anaplasma platys***	13% (8.2, 19.1)	8% (4.3, 13.3)	0% (0, 3.6)	0% (0, 3.6)
***Mycoplasma haemocanis***	17.3% (11.8, 24)	14.2% (9.2, 20.5)	1% (0.03, 5.4)	12% (6.4, 20)

**Table 4:** The occurrence of co-infections with canine TBD by city

Pathogen species	Delhi (n = 57)	Mumbai (n = 44)
**B+E**	7%	0%
**B+H**	7%	13.6%
**B+A**	1.8%	0%
**E+H**	28%	36.4%
**E+A**	7%	4.5%
**E+M**	14%	2.3%
**H+A**	3.5%	4.5%
**H+M**	5.3%	13.6%
**B+E+H**	0%	4.5%
**B+H+A**	0%	2.3%
**B+H+M**	1.8%	2.3%
**E+H+A**	5.3%	0%
**E+H+M**	8.7%	9.2%
**E+A+M**	3.5%	0%
**H+A+M**	1.8%	4.5%
**E+H+A+M**	5.3%	2.3%

B: *Babesia vogeli*

E: *Ehrlichia canis*

H: *Hepatozoon canis*

A: *Anaplasma platys*

M: *Mycoplasma haemocanis*

### Risk factor analysis

There was a significant relationship between location and canine TBD infection (χ^2 ^124.5, df = 3, p < 0.01), which in turn was highly correlated with the presence of ticks on dogs (p < 0.01). Dogs in Delhi and Mumbai were more likely to be infected with ticks and TBD pathogens compared to those from Ladakh and Sikkim. Dogs infected with one or more canine TBD pathogens had a lower PCV (average 29.7%) compared to non-infected dogs (average 35.8%, p < 0.01). Multivariate risk factor analysis (R^2 ^= 0.197) revealed that that dogs infested with ticks were 3.3 (95% CI:2.2, 4.8) times more likely be PCR positive for at least one or more canine TBD pathogen than dogs without tick infestation (p < 0.01), that neutered dogs were 1.9 (95% CI:1.1, 3.4) times less likely to be PCR positive for canine TBDs compared to intact dogs (p = 0.02) and that dogs from refuges were 2.3 (95% CI:1.3, 3.9) times less likely to be PCR positive for canine TBDs compared to stray dogs (p < 0.01). No significant association with age, sex or body score condition with infection was found for any of the parasites.

## Discussion

Despite previous single case reports, to the authors' knowledge this study is the first systematic investigation of the prevalence and diversity of canine TBDs in the regions of Delhi, Mumbai, Sikkim and Ladakh in India using both microscopy and molecular techniques. The study has provided interesting new information about canine TBD, but further investigation using larger numbers of dogs from more localities is necessary in order to gain a truly comprehensive understanding of the distributions of these diseases in India.

Unsurprisingly, the occurrences of both ticks and TBDs in dogs were shown to be higher in Delhi and Mumbai compared to Ladakh and Sikkim. This most likely reflects their different climates, with the former pair being subtropical and tropical, respectively, compared with Ladakh which at significant altitude (3000 m) is arid and dry, and Sikkim which enjoys a more temperate climate. The genus *Rhipicephalus *was found to be the most common tick present in this study followed by *Haemaphysalis*. In this study, ticks were identified morphologically to genus level only. Interestingly, *Haemaphysalis *ticks were identified only in Sikkim, in just over half of the dogs, which again most likely reflects the prevailing climatic conditions of the region that are more suitable ecologically for this genus [42-44] than the other hotter or drier areas of India where sampling was performed. *Haemaphysalis *ticks have been reported previously in the rural highland areas of India such as Jammu Kashmir, Himanchal Pradesh and Arunanchal Pradesh [44], and a study in Japan also revealed that dogs in rural areas carried more *Haemaphysalis *ticks. In contrast *Rhipicephalus *is often associated with dogs in urban areas [45], which is reflected by our findings that *Rhipicephalus *was more common in urban Mumbai and Delhi, compared to rural Sikkim.

In this study *H. canis *was the most common canine TBD pathogen found infecting dogs in India followed by *E. canis*, *M. haemocanis *and *A. platys*. This finding probably reflects the wide geographical distribution of their vector, *R. sanguineus *[46]. In contrast, *B. vogeli *and *B. gibsoni *were detected in fewer dogs. Although it is known that infection with either of these pathogens can result in severe and fatal disease, they can remain clinically undetectable in chronically infected dogs due to very low and often intermittent parasitaemias. Infection with these pathogens may not be apparent or diagnosed until such animals are immunocompromised by unrelated disease or by iatrogenic drug administration or following splenectomy [2,47].

Since the advent of molecular diagnostic testing, it has become increasingly apparent that co-infections of canine TBD are common in other regions of the world [5,22,28,48,49] and this study strongly suggests that the same is true in dogs in India. A recent experimental study reported that co-infection with *A. platys *and *E. canis *can influence various pathophysiological parameters in dogs [24] and supports the notion that multiple infections by canine TBD pathogens may lead to variable and sometimes unexpected clinical outcomes in individuals [5,22,28,48,49]. This is of significant clinical importance as multiple infections in the same host may go undiagnosed, especially if conventional methodology is used, thus frustrating attempts by the veterinary practitioner to adequately treat the individual. Whilst the diagnosis of these diseases is still challenging, a greater awareness of the possibility of canine TBD co-infection is necessary, particularly if poor or partial response to treatment targeting a single agent is observed.

In this study, the prevalence of TBD pathogens in dogs was shown to be positively correlated with the presence of ticks on the animal. However, in addition to this, TBD were less likely to infect dogs from refuges and in those animals that were neutered, even after adjusting for the presence of ticks. This implies that other possibly host- and environmental factors may play an important role in the epidemiology of TBD in dogs. Dogs housed in shelters (refuge dogs) and fed nutritious diets are likely to have a more robust immune status than free roaming strays. Similarly, the hormones oestrogen and testosterone are known to influence the outcome of infectious diseases and this has been widely discussed with respect to parasite infections in humans and non-primate hosts [50,51]. For example, one study reported that the prevalence of intestinal parasites was higher in male dogs and that gonadectomy decreased the likelihood of parasitism in both male and female dogs [52]. In addition to potentially controlling the dog population, de-sexing animals may therefore have a beneficial immuno-protective role for the canine TBD.

In a finding that is now well recognised in epidemiological surveys, the molecular techniques used in this study were shown to be highly sensitive compared to microscopic examinations. Examining stained blood smears is time consuming and requires some level of technical expertise. Often it is not very rewarding as the pathogen is either absent or present in very low numbers; intermittent low parasitaemia is a feature of chronic canine TBD infection [53,54] and poses a significant problem when trying to detect carrier individuals. Thus, the negative findings by microscopic examination in this study were not surprising since none of the dogs showed any evidence of clinical signs. However, blood smear examination remains the simplest and most accessible diagnostic test for veterinarians, to use and is reasonably sensitive during acute, clinically significant infections. Molecular and serological techniques are more useful for detecting chronic and subclinical infections, and are ideally suited to epidemiological investigations as reported here. Despite numerous efforts to optimize PCR screening, because of the nature of these pathogens, a negative result should be interpreted with caution. The combination of haematology, cytology, serology and molecular diagnoses is needed to finalise any screening process to avoid misdiagnoses.

## Conclusion

At least 6 species of canine tick-borne pathogens are present in India. In this study the most prevalent canine TBD pathogen was *Hepatozoon canis*, and *Rhipicephalus *ticks were the most common arthropod vectors identified in Delhi and Mumbai. PCR is more sensitive in detecting blood pathogens compared with microscopic blood film examination. Co-infections between pathogens are common in dogs in India and this warrants increased awareness among veterinary practitioners.

## Competing interests

The authors declare that they have no competing interests.

## Authors' contributions

PAMAR was involved in all phases of the study, including sampling and data collection, laboratory work, data analysis, intellectual interpretation, and writing the manuscript. RJT designed the study project, supervised the study, and was involved in sampling, field data collection, intellectual interpretation and critical revision of the manuscript for publication. PJI, MG and GTC supervised the study and were involved in intellectual interpretation and critical revision of the manuscript for publication. All authors read and approved the final manuscript.
